# Sarcomatoid Carcinoma of the Lung With Gastrointestinal Metastasis Causing Small Intestinal Obstruction: A Rare Case of Acute Surgical Emergency

**DOI:** 10.7759/cureus.38414

**Published:** 2023-05-01

**Authors:** Moses Amarjothi Joacquim, Nadeem Bhat, Nikhil Kulkarni, Andrew Coup, Sridhar Dharmavaram

**Affiliations:** 1 Surgery, United Lincoln National Health Service (NHS) Trust, Lincoln, GBR; 2 General Surgery, United Lincoln National Health Service (NHS) Trust, Lincoln, GBR; 3 General Surgery, Lincoln County Hospital, Lincoln, GBR; 4 Pathology, United Lincoln National Health Service (NHS) Trust, Lincoln, GBR; 5 General and Colorectal Surgery, United Lincoln National Health Service (NHS) Trust, Lincoln, GBR

**Keywords:** sarcomatoid cancer lung, small bowel obstruction, immunohistochemistry, gastrointestinal metastasis, lung cancer

## Abstract

Pulmonary sarcomatoid carcinomas (PSCA) are a rare subset of Non-Squamous-Cell Lung Carcinoma (NSCLC), the most common pathological subtype of lung cancers which are the most common malignancy in the world. These rarest of rare tumours may have gastrointestinal metastasis and present as an acute abdominal surgical emergency with bowel obstruction or bleeding, as in our patient, who did not have a diagnosis of lung cancer till then. Use of novel immunohistochemistry markers like thyroid transcription factor-1 (TTF-1) may help diagnose the site of the primary tumour with accuracy in this clinical setting. Further evaluation of these patients with positron emission tomography (PET) scans helps determine the tumour burden and degree of dissemination. The aim of this article is to emphasize this rare presentation which may be unsuspected as a cause of bowel obstruction by surgical teams on the acute surgical take due to its rarity. It is vital that nihilism is avoided in these patients as a subset of these patients can still be offered curative metastectomy and treatment of the primary tumour with reasonable outcomes.

## Introduction

Sarcomatoid tumours are a rare subtype of poorly differentiated Non-Squamous-Cell Carcinoma of the Lung (NSCLC), the most common broad pathological subtype of lung cancers [1]. These tumours may rarely present as a surgical emergency, causing a diagnostic challenge to clinicians as this aetiology is usually not suspected by surgeons in the usual spectrum of an acute abdominal bowel obstruction.

## Case presentation

**Figure 1:**
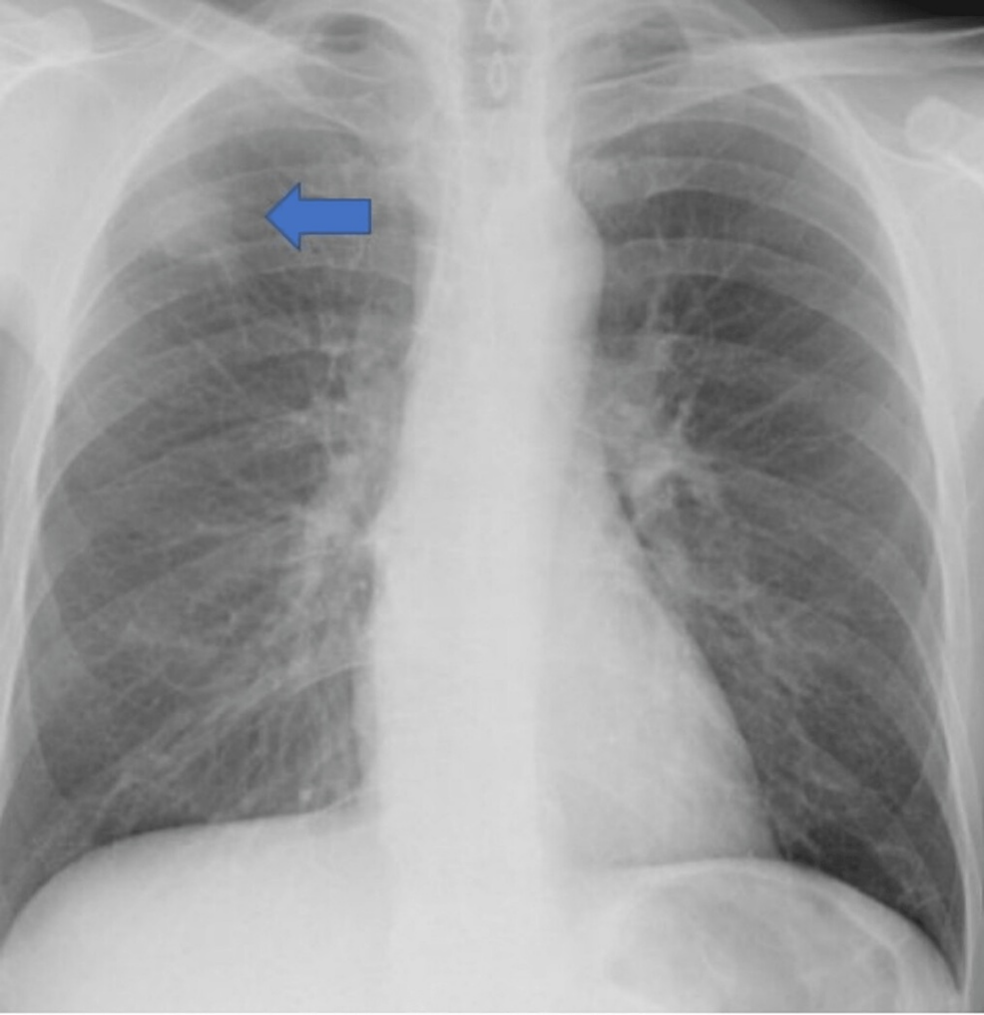
Plain X-ray of chest The image shows the pulmonary nodule in the right upper lung lobe (blue arrow).

A 50-year-old male patient, heavy smoker for 33 years, presented with features of bowel obstruction including abdominal pain, nausea and vomiting for one day. He had history of ischemic stroke for which he was started on antiplatelet (clopidogrel) in the medical ward previously before discharge. An incidental X-ray finding of pulmonary nodule was found during inpatient admission and he was planned for subsequent outpatient CT chest and PET scan before discharge from the ward (Figure 1).

**Figure 2:**
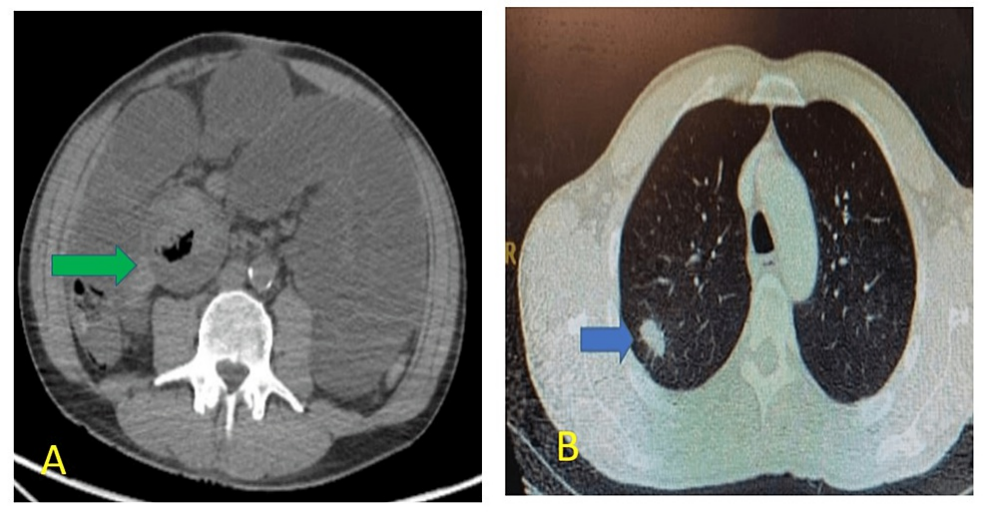
Computer tomography images (A) CT abdomen showing concentric thickening of the jejunal loop (green arrow) along with dilated bowel loops. (B) CT lung showing lung primary tumour in the right upper lobe of the lung (blue arrow).

The patient presented the next day after discharge to the surgical team with features of intestinal obstruction including abdominal pain, nausea and bilious vomiting which necessitated an urgent CT scan of the chest, abdomen and pelvis. This revealed an irregular thickening in the jejuneum with upstream small bowel dilation up to 4.8 cm and enlarged mesenteric lymph nodes (Figure 2A). In addition, a 2 cm lesion in the upper lobe of the right lung with a small 9 mm right hilar lymph node was noted on CT chest correlating with the incidental pulmonary nodule on X-ray chest (Figure 2B). Routine blood investigations revealed a triad of severe leucocytosis (29.8 x 103), anaemia and thrombocytosis.

**Figure 3:**
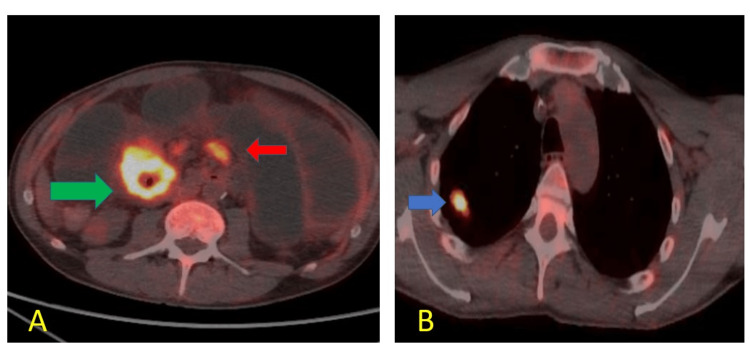
PET scan images Positron emission tomography (PET) scan showing (A) hypermetabolic concentric jejunal metastasis with luminal narrowing (green arrow) and multiple mesenteric lymph nodes (red arrow); (B) the pulmonary lesion is seen in the right upper lung lobe (blue arrow).

Subsequent PET CT scan done revealed the same proximal intestinal obstruction in the jejuneum which was noted on the previous CT scan with thickening for about 6 cm and a standardized uptake value (SUV) max uptake of 25.6. Another nonobstructive area of increased uptake in the ileum and multiple mesenteric lymph nodes were seen in addition to the above (Figure 3A). This was seen along with the primary tumour in PET scan (Figure 3B).

**Figure 4:**
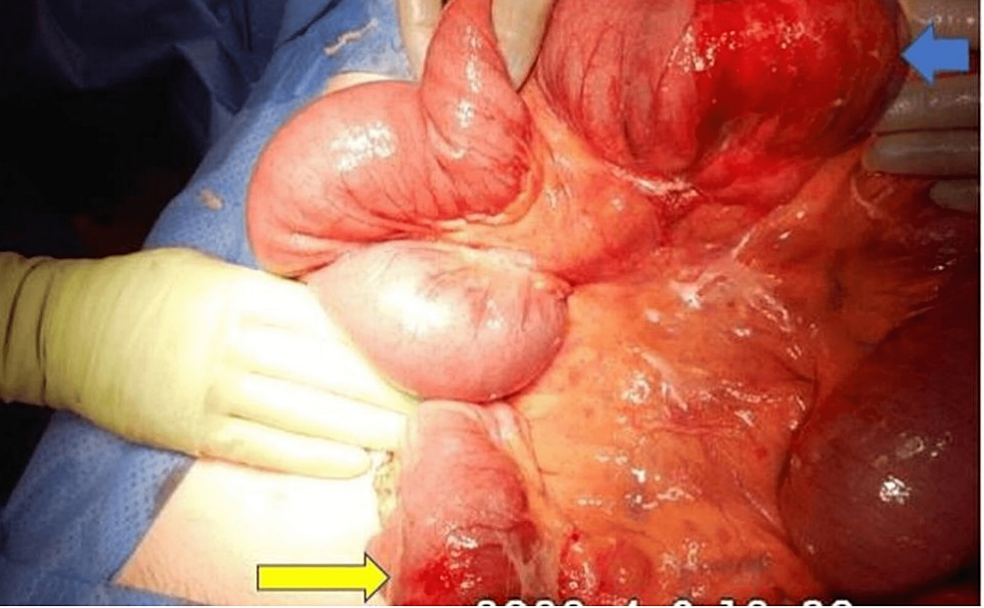
Intraoperative image Dilated jejunal loops with jejunal thickening (blue arrow) and ileal involvement (yellow arrow).

The clinical condition of the patient deteriorated after presentation which necessitated an emergency laparotomy with small bowel resection of both affected segments in the jejuneum and ileum with adequate lymphadenectomy (Figure 4). Primary anastomosis of the resected bowel was deferred as the patient was on blood thinner medicines. Though a high output of jejunal stoma was recognised as a potential problem postoperatively, it was considered the safest option considering the patients general condition and intraoperative situation.

**Figure 5:**
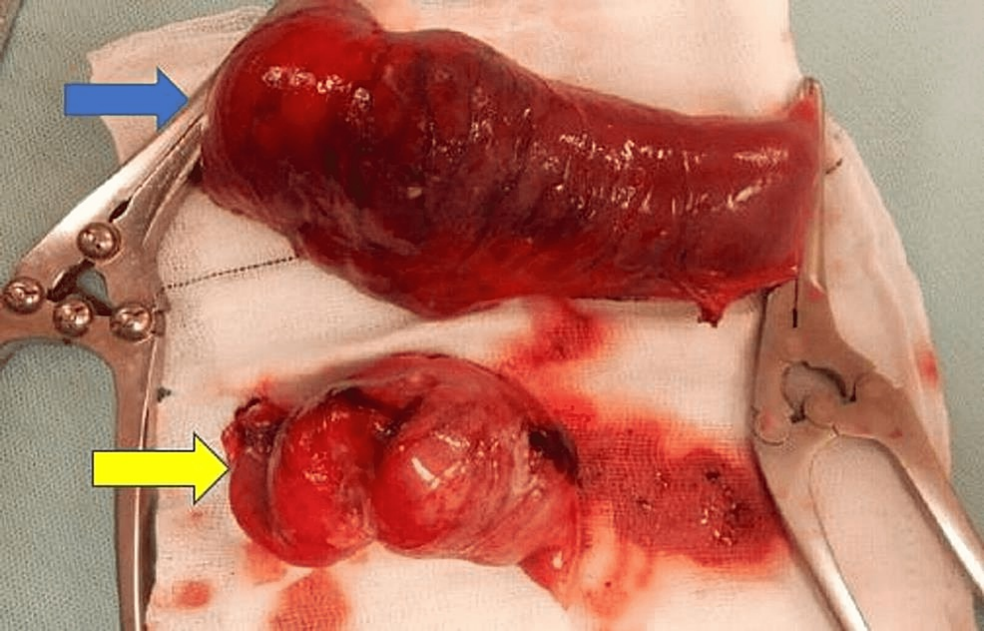
Resected specimens Resected jejunal segment (blue arrow) and ileal segment (yellow arrow).

**Figure 6:**
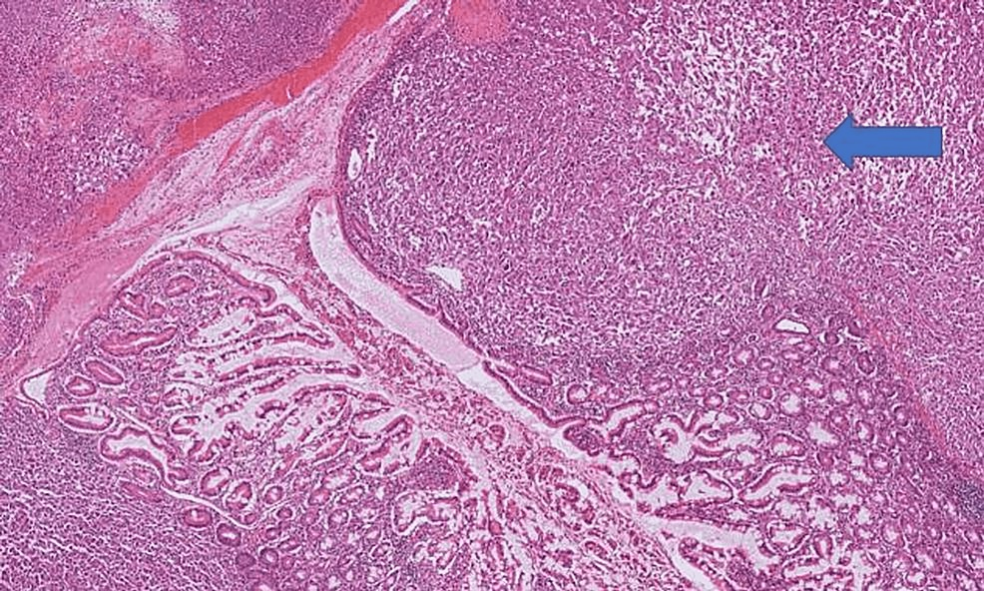
Histology image H&E 200X. Histology of resected small bowel specimen showing normal mucosa overlying tumour cells with plump eosinophilic cells, pleomorphic nuclei, and numerous mitotic figures (blue arrow).

The resected specimens revealed two separate tumours in the small bowel, both with similar morphology (Figure 5). On histological testing, the tumours were found to be composed of plump eosinophilic spindle cells, with pleomorphic nuclei and numerous mitotic figures. (Figure 6) The overlying small bowel mucosa in the specimen was normal.

**Figure 7:**
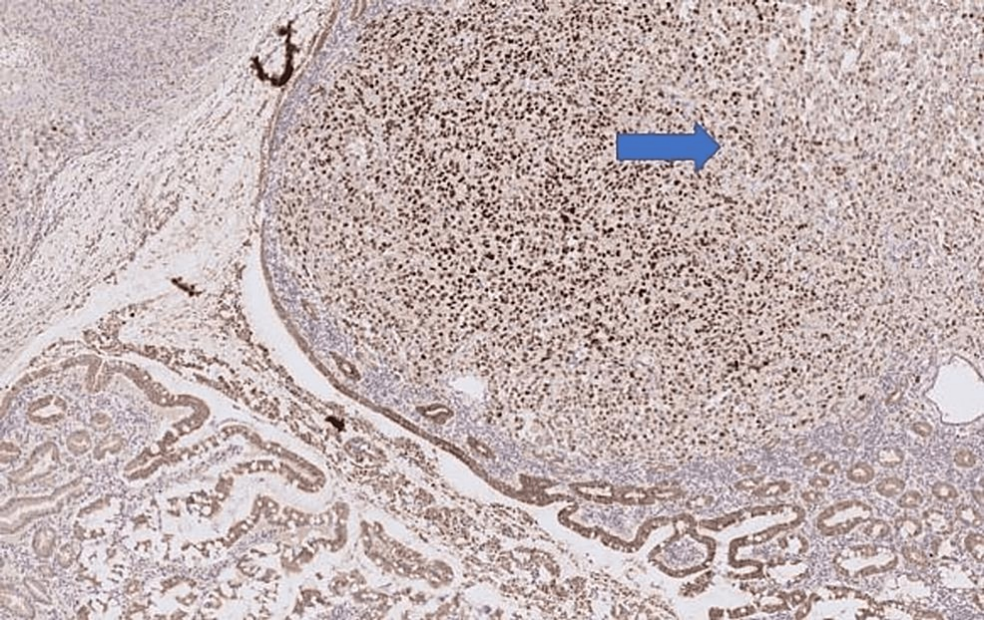
Immunohistochemistry image 1 IHC X200. TTF-1 expression in the tumour cells (nuclear staining) of the resected small bowel specimen (arrow).

**Figure 8:**
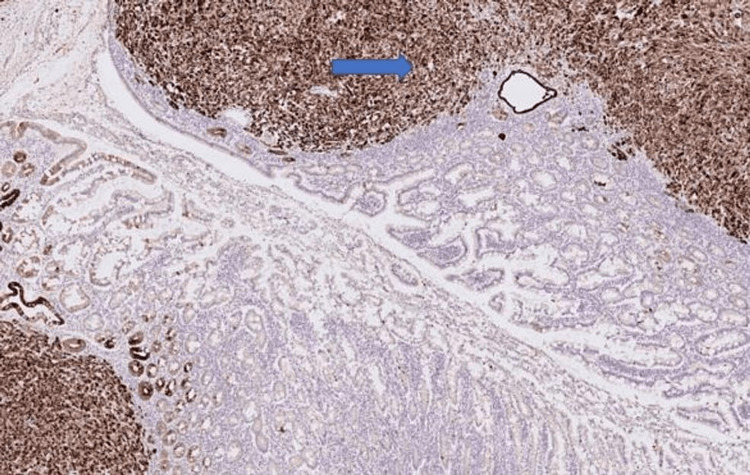
Immunohistochemistry image 2 IHC X200. Tumour cells showing strong CK7 expression in the resected small bowel specimen (arrow).

**Figure 9:**
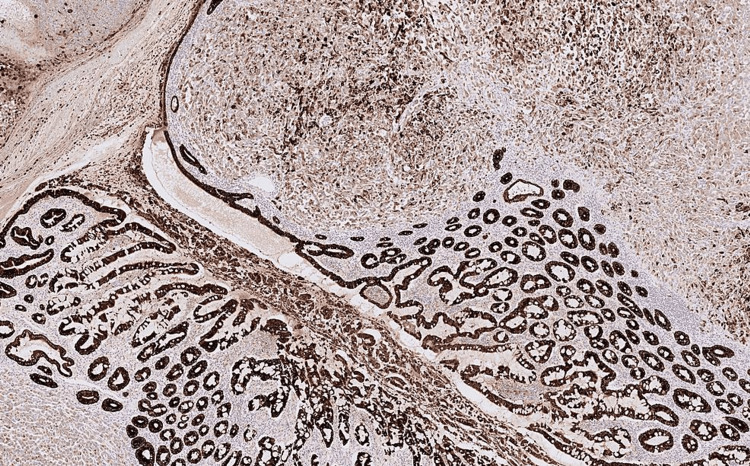
Immunohistochemistry image 3 IHC X200. Tumour cells showing strong cytokeratin (AE1/3) expression in resected small bowel specimen.

Immunohistochemistry showed strong expression of AE1/3 in the tumour cells, together with CK7, p63 and TTF-1 (Figures 7-9 ). They were negative for CD117, DOG1, CK20, Desmin, MelanA and S 100.

**Figure 10:**
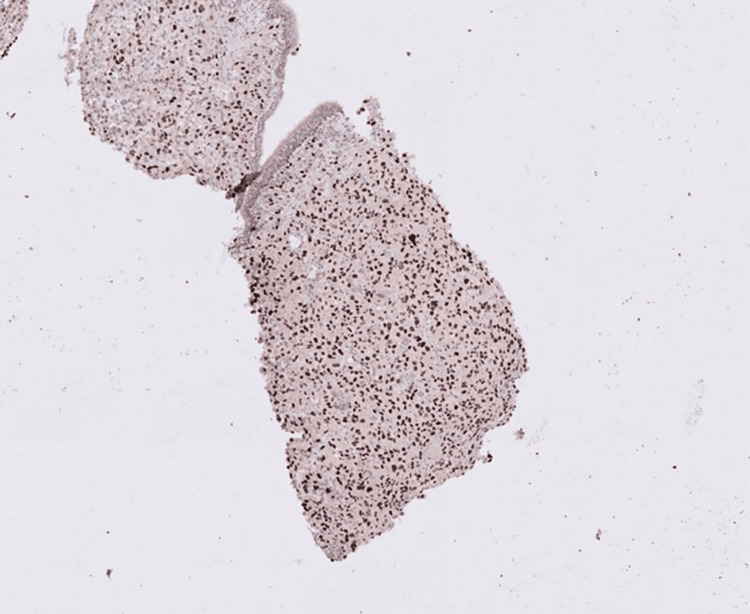
Lung core biopsy image IHC X 200. Lung core biopsy image showing tumour cells with TTF- 1 expression.

This confirmed a diagnosis of sarcomatoid carcinoma from the lung and excluded the possibilities of primary intestinal pathologies including gastrointestinal stromal tumour and leiomyosarcoma. The combination of multifocal submucosal tumours with normal overlying mucosa favoured metastatic disease rather than small bowel primary tumour and TTF-1 expression raised the possibility of metastases from a lung primary tumour. A subsequent biopsy of the right lung confirmed a primary lung carcinoma with similar morphology to that seen in the bowel (Figure 10).

The patient was managed with reversal of stoma in the jejunum done one month later and is doing well one month after discharge and is currently on maintenance novel chemotherapeutic agent pembrolizumab after prior four cycles of combination carboplatin, pemetrexed and pembrolizumab with minimal adverse effects. There is an impressive 40 percent reduction in the size of the primary tumour in comparison to the maximum tumour size after the systemic chemotherapy. It is to be noted that this partial response was seen even as the analysis of the primary tumour revealed PD1 negative, EGFR negative, ALK negative, ROS negative, BRAS negative, K-ras exon 2 mutation positive and K-ras G12 c mutation negative statuses. He is also planned for palliative radiotherapy (20 Gray in five fractions) to the lung.

## Discussion

Pulmonary sarcomatoid carcinoma (PSCA) is a universally accepted term for a group of infrequent, (0.4% of total lung cancers), poorly differentiated, highly metastatic, subset of NSCLCs that contain a component of sarcoma-like differentiation contributing at least 10 percent of the total cell type [2]. Romano et al. described these tumours which occur mainly in elderly smoking men [3]. Aggressive tumour biology as manifested by rapid growth, marked central coagulative necrosis or cavity formation, early vascular, lymph node involvement and high metastatic potential contribute to poor prognosis even with pN0 disease [4,5]. Most gastrointestinal tract (GIT) metastasis of lung cancers including sarcomatoid carcinoma are asymptomatic. Late-stage serious complications, include intussusception leading to obstruction (most common), perforation or bleeding. PET-CT plays a role in systemic evaluation of extra-GIT metastases even in rare areas like epiglottis [6].

Differentials like gastrointestinal stromal tumours are excluded by lack of CD117, DOG-1 and metastatic melanoma by Melan-A and S100. TTF-1 is an important marker of lung, thyroid carcinomas and by most small cell carcinomas irrespective of site of origin [7]. Careful patient selection for surgery on primary and metastatic sites can result in a favourable outcome. Surgical treatment with resection of small bowel metastasis with or without restoration of bowel continuity is required or is the only possible treatment and can be offered as long as the patient’s physical condition can tolerate the proposed procedure. Long term survival is possible if the metastatic lesions are resectable with low tumour burden along control of the primary tumour which may include surgery, radiotherapy or use of novel systemic agents like in our case.

The management of these previously untreatable and lethal cancers has been revolutionised with the addition of novel immunotherapeutic agents like pembrolizumab, a programmed death ligand (PD-L1) inhibitor. In an updated analysis of the novel KEYNOTE-189 trial, a large randomised control trial involving pemetrexed-platinum based chemotherapy with or without the addition of pembrolizumab, the addition of pembrolizumab in addition to the chemotherapy has demonstrated substantially improved median overall survival (OS; 22 months vs 10.7 months) and median progression free survival (PFS) (9 months vs 4.9 months) in metastatic nonsquamous NSCLC and with manageable safety and tolerability profile [8]. This effect is surprising regardless of PD-L1 receptor expression or liver/brain metastases, as seen in our patient who is PD-L1 negative with resected bowel metastasis.

## Conclusions

Small bowel metastasis with primary tumour in the lungs is rare and can present as a surgical emergency with acute intestinal obstruction or bleeding, as exemplified by the patient described here. Evaluation of the chest, in addition to the abdomen, is necessary for diagnosis. Novel immunohistochemistry markers like TTF-1 can help identify the site of the primary tumour in this setting along with PET scans to diagnose the degree dissemination. This helps prognosticate patients with low tumour burden who can be managed by curative metastatic resection and good primary tumour control, as in our aforementioned case.
